# Mechanisms Causing Acantholysis in Pemphigus-Lessons from Human Skin

**DOI:** 10.3389/fimmu.2022.884067

**Published:** 2022-05-20

**Authors:** Desalegn Tadesse Egu, Thomas Schmitt, Jens Waschke

**Affiliations:** Chair of Vegetative Anatomy, Institute of Anatomy, Faculty of Medicine, Ludwig-Maximilians-Universität Munich, Munich, Germany

**Keywords:** pemphigus, signaling, desmosomes, ultrastructure, *ex vivo* skin model, electron microscope

## Abstract

Pemphigus vulgaris (PV) is an autoimmune bullous skin disease caused primarily by autoantibodies (PV-IgG) against the desmosomal adhesion proteins desmoglein (Dsg)1 and Dsg3. PV patient lesions are characterized by flaccid blisters and ultrastructurally by defined hallmarks including a reduction in desmosome number and size, formation of split desmosomes, as well as uncoupling of keratin filaments from desmosomes. The pathophysiology underlying the disease is known to involve several intracellular signaling pathways downstream of PV-IgG binding. Here, we summarize our studies in which we used transmission electron microscopy to characterize the roles of signaling pathways in the pathogenic effects of PV-IgG on desmosome ultrastructure in a human *ex vivo* skin model. Blister scores revealed inhibition of p38MAPK, ERK and PLC/Ca^2+^ to be protective in human epidermis. In contrast, inhibition of Src and PKC, which were shown to be protective in cell cultures and murine models, was not effective for human skin explants. The ultrastructural analysis revealed that for preventing skin blistering at least desmosome number (as modulated by ERK) or keratin filament insertion (as modulated by PLC/Ca^2+^) need to be ameliorated. Other pathways such as p38MAPK regulate desmosome number, size, and keratin insertion indicating that they control desmosome assembly and disassembly on different levels. Taken together, studies in human skin delineate target mechanisms for the treatment of pemphigus patients. In addition, ultrastructural analysis supports defining the specific role of a given signaling molecule in desmosome turnover at ultrastructural level.

## Introduction

Epithelial cells are tethered to one another by different types of intercellular adhesion complexes. Desmosomes form the core of these junctional complexes and provide resilience to tissues that constantly encounter mechanical forces (1, 2). They consist of members of three protein families, the cadherin superfamily which comprises two subclasses of Ca^2+^- binding transmembrane proteins, the desmogleins (Dsg) and desmocollins (Dsc), each with distinct isoforms, Dsg1-4 and Dsc1-3, respectively (3); armadillo protein family including the plakoglobin and plakophilins 1-3 (Pg and Pkp 1-3); as well as the plakin family member desmoplakin (Dp) also are among the core components of desmosomes (4). Besides, plectin, which also is a member of the plakin family, is involved in desmosome organization by crosslinking the peripheral intermediate filament and actin cytoskeleton (5).

The essential function of desmosomes is compromised under diseased conditions such as pemphigus. Pemphigus is a rare group of autoimmune diseases affecting the skin and oral mucosa but less frequently involves mucous membranes of other organs such as the eyes and genitals (6). Based on immunological and histological characteristics, three major phenotypes of pemphigus are recognized; pemphigus vulgaris (PV), pemphigus foliaceus (PF), and paraneoplastic pemphigus (PNP) (7). PV is caused by autoantibodies which primarily target Dsg1 and Dsg3 (8–10). It is characterized by suprabasal splitting in the epidermis and/or oral epithelia. PF lesions are confined to the epidermis and are triggered by anti-Dsg1 autoantibodies which results in erosions and flaccid blisters in the superficial epidermis, mainly in the granular layer (11). PF is most frequent in some countries in South America and North Africa due to the presence of an endemic form of the disease affecting mainly young adults (12). PNP is characterized by mucocutaneous lesions with diverse clinical presentations including suprabasal blisters and interface dermatitis (13, 14). The presence of neoplasms associated with tissue lesions is the main distinguishing feature of PNP from PV and PF (15). PNP is caused by autoantibodies directed against a variety of autoantigens including Dsg1, Dsg3, and also Dsc1and Dsc3 (16) as well as plakin family proteins (17). Other very rare variants of pemphigus include pemphigus vegetans, pemphigus erythematosus, and pemphigus herpetiformis (7).

Available treatment options mainly focus on modulation of the immune system such as depletion of autoantibody-producing B cells as well as non-specific immunosuppressive agents including corticosteroids and others with associated side effects emanating from long-term administration (18). Besides, chemical inhibitors such as rilzabrutinib, a potent inhibitor of Bruton tyrosine kinase (BTK) (19), has been reported as a promising therapeutic strategy at phase II clinical trial (20). Because of an unmet medical need to treat patients until autoantibody formation can be suppressed, current research focuses on devising novel therapeutic approaches including suppressing specific signaling pathways involved in pemphigus pathogenesis (21). Therefore, in this mini-review we will discuss the role of signaling pathways, which have been delineated to ameliorate acantholysis in several models of PV *in vitro, in vivo* and *ex vivo* (22), for the regulation of desmosome ultrastructure as revealed by transmission electron microscopy. We will highlight the significance of a human skin organ model as a useful tool to understand the underlying pathophysiology of pemphigus diseases by providing a physiological relevant near-to-patient situation.

## Desmosomes

**Figure 1:**
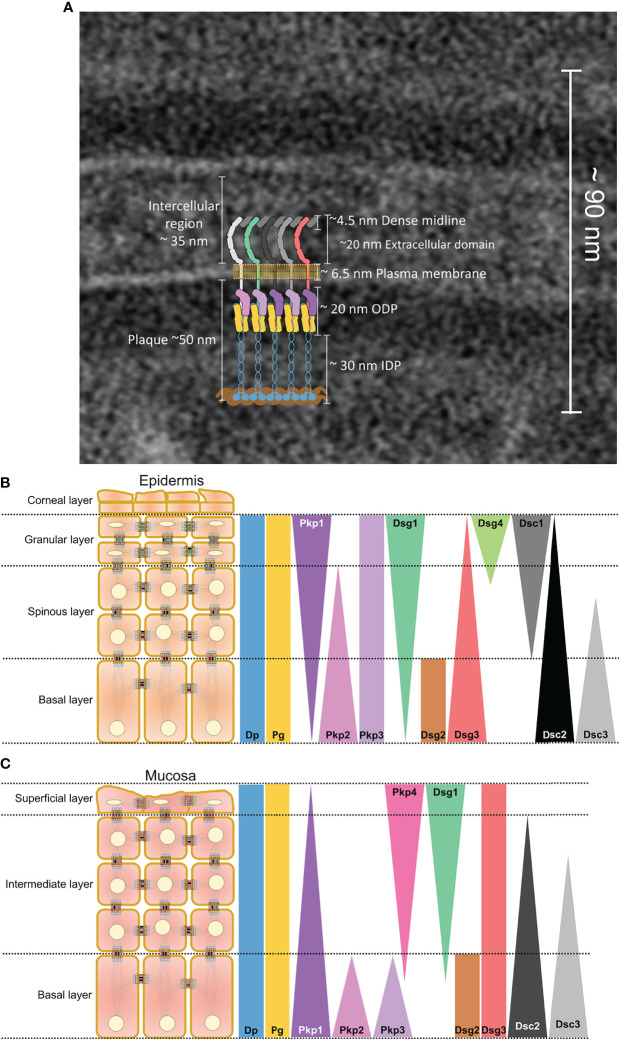
**(A)** Electron micrograph showing single desmosome in a healthy skin with a superimposed schematic representation depicting molecular structure of a desmosome. Colour coding of each single molecule is the same as those represented in **(B, C)**. A schematic representation of the distribution of desmosomal proteins along the different layers of **(B)** epidermis and **(C)** mucosa. Small schematic desmosomes are colour-coded the same as the bars representing the distribution.

Desmosomes are recognized in electron micrographs by spatial distribution of electron dense plaques of varying densities identified as outer dense plaques (ODP), inner dense plaques (IDP), and extracellular core (EC) (23, 24) (**Figure 1A**). The components of these plaques were identified using immunoelectron microscopy (25). A more precise localization of the terminal domains of the main desmosomal proteins has been achieved using the quantitative immunogold method (23). Accordingly, the intracellular core of Dsg and Dsc, Pg and Pkp, as well as the N terminus of Dp constitute the ODP, whereas the N domain of Dp forms the IDP (23) and anchors the desmosomal plaques to the intermediate filament cytoskeleton (26).

The different desmosome proteins exhibit different distribution patterns across the different layers of epithelia as a function of tissue type and differentiation status (27–29) (**Figures 1B, C**). In the epidermis, Dsg2 and Dsg3 are predominantly expressed in basal keratinocytes, whereas Dsg1 and Dsg4 are localised to the differentiated suprabasal layers (27). Dsg1together with Dsc1 shows an inverse distribution gradient with Dsg3 and Dsc3 across the suprabasal compartments in which Dsg1 and Dsc1 are strongly expressed as the cells differentiate and stratify. Dsg2 and Dsc2 are ubiquitously present in all desmosomes bearing tissues including the heart and simple epithelia (30). Dsg2 is present in the basal cell layer of oral mucosa (31, 32) and neonatal epidermis but restricted to hair follicles in adult human epidermis (33). Moreover, Dsg3 is the dominant desmoglein present in mucosa, whereas Dsg1 is distributed in all layers except the proliferating basal layer but at low levels (27, 32, 34, 35).

Through their extracellular N- domains, desmosomal cadherins are known to make *cis* and *trans* interactions with their counterparts on the same or adjacent cells, respectively, to form knot-like structures with desmosomes (36, 37). Although *cis* interactions are thought to be weaker, both mechanisms synergistically contribute to the formation of a stable adhesion complex (38). Interaction between similar cadherins (homophilic) as well as between different cadherin subclasses (heterophilic) has been reported (4, 39–41). Recent studies using bead assay identified heterophilic trans-interactions between Dsg1/Dsc1 and Dsg3/Dsc3 as the strongest and dominant form of adhesion in desmosomes (42, 43). In line with this, in atomic force microscopy (AFM) experiments Dsg2/Dsc2 formed a more stable dimer with a prolonged bond lifetime (44). In addition, homophilic interaction of Dsg1, Dsg3, and Dsc3 have been demonstrated as well (45–49). For Dsg2, it was reported recently that the interaction modes with desmosomal cadherins Dsg2 and Dsc2 differ from binding events with classical cadherins E-cadherin and N-cadherin (50). Moreover, heterophilic interaction of Dsg2, which is up-regulated in PV, with Dsg3 was proposed as compensatory mechanism because it was found to be resistant to autoantibody-induced steric hindrance (51). Taken together, both homo- and heterophilic interactions of desmosomal cadherins contribute to tissue integrity in epithelia (52, 53).

### Other Desmosome-Related Diseases

Desmosomes have been studied extensively in recent years in consequence of diverse disease phenotypes including genetic and acquired diseases as well as autoimmune or microbial-mediated diseases which weaken intercellular adhesion (54).

Gene mutations involving obligate desmosomal proteins such as Dsg, Dsc, or Dp may bring about a wide spectrum of genetic diseases of the skin and other tissues in which the proteins are strongly expressed (55–57). In the skin, mutations of Dsg1 have been shown to cause severe dermatitis, multiple allergies, and metabolic wasting (SAM syndrome), in which loss of Dsg1 besides epidermal splitting causes a profound alteration of the epidermal barrier as well as the immune system, as shown in Dsg1-deficient animal models (58–61). In humans, mutations in desmosomal components are also associated with heart diseases. Mutations in Dsg2 and Dsc2 are known to cause arrhythmogenic right ventricular cardiomyopathy whereas those in Pg and Dp cause cardiomyopathy as well as various cardio-cutaneous syndromes (62–65). Some aspects in the pathophysiology of arrhythmogenic right ventricular cardiomyopathy may be similar to pemphigus due to common aspects in the structure and regulation of desmosomal contacts in cardiac intercalated discs and desmosomes of the epidermis (66). Moreover, mutations in desmosomal proteins have been shown to affect skin appendages. For example, homozygous and heterozygous mutations of Dsg4 have been reported to be associated with a spectrum of disease phenotypes such as hypotrichosis and monilethrix (67–69). Similarly, genetic alteration in Dsc3 was identified as the underlying cause for hair loss and vesicle formation in skin (70). Mutations in Dsg3, although not known in humans so far, caused diverse clinical features, including severe skin and mucosal lesions and hair loss as in squeaking (sqk) phenotype mice (71).

Microbial and viral agents are among the extrinsic factors that alter expression of desmosomal proteins (72). Exfoliative toxin A produced by staphylococcus bacteria is known to target Dsg1 and proteolyzes its adhesive ectodomain resulting in a PF-like lesion as in a bullous impetigo or staphylococcal-scalded skin syndrome (73) An adenovirus known for affecting the epithelial lining of the respiratory and urinary tracts was identified to bind to Dsg2 destabilizing cell-cell attachment (74). Studies in other acquired diseases such as cancer have revealed dysregulation of desmosomal proteins in tumor cells. For example, Dsg1 is downregulated in squamous cell carcinoma whereas Dsg3 is upregulated in head and neck carcinoma (57). Moreover, some molecular mechanisms underlying desmosome dysregulation in cancer cells have been reported (75, 76). Finally, alterations in desmosome ultrastructure have been detected in patients suffering from inflammatory bowel disease (77, 78). Because animal models deficient for Dsg2, Dsc2, and Dp have been shown to have intestinal epithelial barrier defects and disturbed wound healing and are prone to colitis (79–82), several lines of evidence indicate that disturbed desmosomal adhesion contributes to the pathogenesis of inflammatory bowel diseases (83). In this respect, a new function of desmosomes has been elucidated as they control tight junction structure and function (84, 85).

## Autoantibody Profiles and Their Roles in PV

Autoantibody profiles in pemphigus patients’ serum dictate the specific disease phenotype manifested (22, 52, 86). Titers of Dsg-specific autoantibodies in pemphigus indicate disease activity as well as progression (87–89). It is known that these autoantibodies consist of both pathogenic and non-pathogenic forms (90, 91) which possess distinct epitopes they preferentially bind to (92, 93). It has been identified that the pathogenic autoantibodies target the EC subdomains of Dsg3 (EC1-3) and cause cell-cell detachment, whereas nonpathogenic antibodies bind to membrane-proximal domains without affecting cell adhesion (94, 95). Skin biopsies from pemphigus patients were examined to identify the tissue- and layer-specific binding of IgG from various pemphigus phenotypes (96, 97). The tissue as well as layer-specific distribution of lesions has been attributed to differential expression of desmosomal proteins among various tissues and across the layers of stratified epithelia (86). According to this hypothesis, anti-Dsg3 reactive antibodies cause suprabasal blistering owing to low expression of Dsg1 in deep layers so that it cannot compensate for Dsg3 as the case in PV. Similarly, anti-Dsg1 autoantibodies cause depletion of Dsg1 in the superficial layers where Dsg3 expression is very low as observed in PF (34, 96, 97). However, this theory has been challenged because it cannot explain why blister formation in PV is restricted to the basal-suprabasal interface. Besides, involvement of other non-Dsg autoantibodies in the disease in some cases or lack of a strong correlation between anti-Dsg titers and disease manifestation in some patients was reported (98–100). In light of this, several non-Dsg antigenic targets, which exhibit a strong autoreactivity to PV sera and with a potential to cause acantholysis, have been identified. These target antigens include cholinergic receptors (101), mitochondrial proteins (102), as well as other desmosomal proteins, such as desmocollins (16, 103–105) and Pkp3 (105), adherens junction protein E-cadherin (106), and others (100). However, the fact that Dsg-specific autoantibodies cause altered distribution followed by internalization of desmosomal proteins and are sufficient to cause skin blistering (107–112) underscores the role of anti-Dsg autoantibodies as a major pathogenic factor in pemphigus. In line with this, immunoadsorption of pathologic autoantibodies from PV sera by the entire EC domains of Dsg1 and Dsg3 abolished the blister-inducing ability of IgG fractions (113, 114).

## Models for Studying Pemphigus

Several experimental models have been established to explore the underlying pathomechanisms of pemphigus. These diverse setups have allowed the characterization of the immunological and molecular mechanisms involved in autoimmune blistering diseases by reproducing some features of the disease as manifested in patients. Moreover, it enabled testing the efficacy of some therapeutic agents in a physiological setup which otherwise would be difficult to undertake in humans (115). The models include cell culture, organotypic tissue culture, animal models mainly mice, and *ex vivo* human skin model.

### Cell and tissue cultures

Isolated cell lines from humans and murine sources have been utilized to investigate the pathogenic effects of pemphigus autoantibodies *in vitro*. The most extensively used epidermal cells are immortalized human keratinocyte (HaCaT cells: Human adult high Calcium low Temperature) (116) and primary human or mouse keratinocytes. Both cells were utilized mainly as monocultures but were also used to construct a three- dimensional (3D) organ culture model representing a stratified and differentiated epidermis (117). Both cell types have pros and cons in terms of originality, reproducibility, cost, and so on (118). The models are vital to assess the level of pathogenicity of autoantibodies derived from PV patients through various functional dissociation assays as well as to determine the effects of different pharmacological mediators in response to the autoantibodies (119–121).

### Mouse model

Animal models are used extensively in medical research. Several mouse models have been developed which rendered great insights into PV pathogenesis (122). Based on the method in which the disease is induced and persistence of clinical features, the models can be identified as active or passive. Passive animal model refers to administration of pemphigus autoantibodies into a healthy animal which results in production of a transient disease phenotype (123, 124). The active models represent the generation of animals which manifest the disease through genetic modification as in Dsg3 or Dsg1 knockout mice (9, 125) or immunization in which the mice actively produce antibodies against Dsg3 (90, 126). In the latter, splenocytes from Dsg3 knockout mice, following immunization with recombinant Dsg3, were transferred into Rag2 knockout mice expressing Dsg3 (126–128). This model is more relevant as it involves active production of anti-Dsg3 autoantibodies similar to the situation in PV patients, whereas Dsg3 knockout mice produce a disease phenotype which is an ultrastructural correlate of only the acute phase of the disease (129).

### Ex vivo model

**Figure 2:**
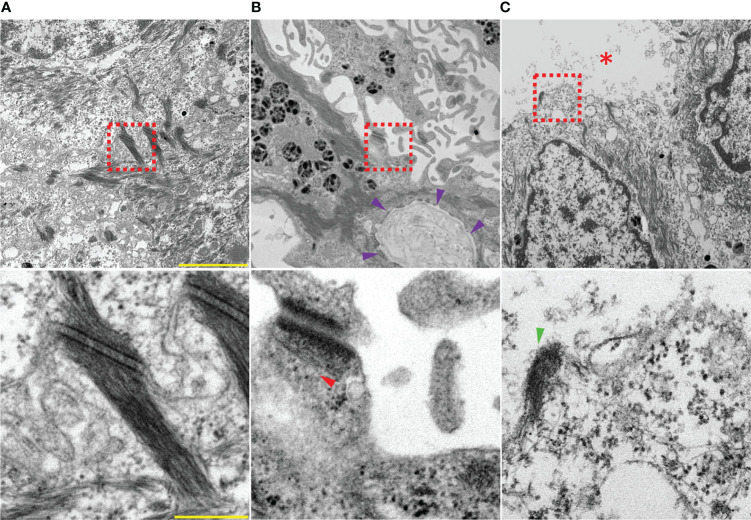
Electron micrographs showing an overview (top) and zoomed in to a single desmosome (bottom) of **(A)** healthy skin injected with IgG from healthy volunteers. **(B, C)** skin injected with PV-IgG showing suprabasal blistering, interdesmosomal widening and altered desmosomes: **(B)** reduced keratin insertion (red arrow head) into a damaged plaque. Note that the distinction between the outer and inner desmosomal plaque is lost after incubation with PV-IgG; violet arrow heads indicate the basement membrane, **(C)** a split desmosome with half plaque (green arrow head), red asterisk indicates blister cavity. Scale bars: 2 µm (top) and 250 nm (bottom).

Organ culture has been an important experimental model for studying pemphigus since the pioneer work done by Schiltz and Michel (130) in which they placed a skin biopsy on a lens paper floating on a liquid medium containing unpurified PV sera. This model is of paramount importance because of the viability of the tissues at optimal time point, 24 h, as well as the potential of the method to reproduce all the major histological and clinical features of the disease. This model stands out to be better than animal models since it enables overcoming the genetic and immunological differences which otherwise would elicit respective species-specific autoreactions (120). Furthermore, it favors the assessment of the pathogenicity of a given autoantibody and helps to correlate to disease activity in patients to develop more specific therapeutic strategies (120, 121). Although organ culture models are not best suited for biochemical studies, they provide a physiologically relevant setup to investigate mechanisms causing altered expression of desmosomal proteins and the resulting acantholysis (131, 132) which closely reflects the human *in vivo* situation (115). Thus, the model is ideal to conduct experiments under controlled conditions with sizeable samples which otherwise are not feasible to apply to human subjects. Therefore, we employed a human skin organ culture model as well as a novel mucosa *ex vivo* model to investigate the role of various signaling molecules in PV pathogenesis (32, 59, 133–136). Large blisters with associated ultrastructural changes in desmosomes including reduction in desmosome density and size as well as formation of split desmosomes and keratin filament uncoupling from desmosomes was observed in samples treated with PV-IgG (**Figures 2A–C**) (135) which shows that the skin model reflects the ultrastructural hallmarks known from pemphigus patients’ lesions (137, 138). Interestingly, the outer and inner plaque could not be differentiated after treatment with PV-IgG, an observation which requires further attention, especially because it has recently been shown that reorganization of the desmosomal plaque occurs during desmosome maturation and it is conceivable that these events may be reverted in pemphigus pathogenesis (139). Also, it must be noted that PV-IgG containing both autoantibodies against Dsg1 and Dsg3 were required for acantholysis whereas mucosal-dominant PV-IgG with autoantibodies against Dsg3 but not against Dsg1 similar to AK23, which is specific for Dsg3, were not sufficient (135). Taken together, the model better reflects the situation in patients compared to mice where high concentrations of anti-Dsg3-specific IgG are sufficient to cause skin blisters (114, 140).

### Mechanisms Causing Acantholysis

Although not fully unraveled, steric hindrance and signaling have been proposed as the major overarching pathomechanisms that drive loss of intercellular contacts downstream of PV-IgG binding to keratinocytes (52, 141). Both mechanisms are believed to be involved, but not strictly independent of each other (57), and the exact chronology of events as well as contribution of each remains unknown.

### Steric Hindrance

Passive transfer of IgG from PV patients or anti-Dsg3 monoclonal antibodies to a healthy neonatal mouse has been shown to induce epidermal blisters (90, 94) which has been reproduced in human skin organ culture, as well (142). These pathogenic immunoglobulins were shown to preferentially target the EC1 and EC2 adhesive regions of Dsg3 (90, 92, 94, 143). The latter has been indicated to predominatly contain epitopes recognised by PV autoantibodies (92, 144, 145). As a result, it has been suggested that PV-IgG autoantibodies directly interfere with the adhesive interaction of Dsg, thereby triggering the initial events leading to acantholysis (90, 127). AFM studies on cell-free surfaces (45, 46, 146) or on living keratinocyte cell surfaces, combined with bead assays, demonstrated direct inhibition of Dsg interaction for Dsg3 but not for Dsg1 (45, 53, 146), but suggested that direct inhibition is not sufficient to cause complete loss of keratinocyte adhesion. In addition, a recent study using bead assays under cell-free conditions showed that PV-IgG and PF-IgG blocked the heterophilic interaction between Dsg3/Dsc3 and Dsc1/Dsc1, respectively (43, 106).

### Role of Signaling

The first signaling pathway to be triggered by pemphigus autoantibodies was PLC-mediated influx of Ca^2+^ (147). Along the years, several studies have shown that steric hindrance alone does not adequately account for acantholysis (45, 111, 146, 148–150), which indicated the involvement of other mechanisms underlying the pathogenic effect of PV-IgG. Phosphorylation and activation of signaling cascades downstream of PV-IgG binding to target antigens in keratinocyte both *in vitro* and *in vivo* has highlighted the crucial role of signaling in PV pathogenesis (151–154). As a result, a great number of signaling molecules that are implicated in PV have been identified and characterized (22, 155). These include mitogen-activated kinases (MAPKs) such as p38MAPK, protein kinase C (PKC), extracellular signal-regulated kinases (ERK1/2), Rous sarcoma-related kinases (Src), phospholipase C (PLC), Epidermal growth factor receptor (EGFR), and other cellular responses that alter adhesive interactions (22, 48, 59, 110, 112, 131, 147, 151, 156–162). For several years, the plethora of signaling mechanisms appeared to be triggered without recognizable hirarchy upon binding of autoantibodies. However, it was shown that signaling molecules such as p38MAPK, PI4K, PLC, and PKC directly bind to desmogleins and that Dsg1 and Dsg3, together with Pg, organize overlapping yet distinct signaling hubs (136, 140, 163). These findings help to explain why autoantibodies against Dsg3 and Dsg1 were observed to cause different sets of signaling responses (48) and led us to propose that the different clinical phenotypes of pemphigus with respect to mucosal and skin involvement, as well as suprabasal versus superficial epidermal blistering, may at least in part be caused by the different signaling profiles observed in PV and PF (22).

### p38MAPK Regulates Autoantibody-Mediated Ultrastructural Alteration of Desmosomes

p38MAPK has been thoroughly characterized due to its essential role in pemphigus pathophysiology. The different isoforms (α, β, γ, δ) display a species-specific expression pattern (164–166) among which the α subtype is the most commonly expressed isoform in adult tissues (165, 167). Activation of p38MAPK was detected in keratinocyte cell cultures treated with PV-IgG and in mouse skin (151). Moreover, p38MAPK was phosphorylated in perilesional skin of PV patients (153, 168) as well as in the skin of Dsg3-deficient mice (169). This signaling molecule has also been shown to be associated with Dsg3 (33, 112, 136, 140), Dsc3 (169), and Dsg1 (136). p38MAPK-mediated Dsg3 internalization followed by depletion from endosomes was also detected in keratinocyte cultures and patient skin (108, 112, 170). Interstingly, previous studies have shown that pharmacologic inhibition of p38MAPK was sufficient to avert cell dissociation *in vitro* (33, 48, 151, 171), rescue membrane-bound as well as cytoskeletal fractions of Dsg3 (112, 172, 173), and reorganize keratin cytoskeleton (49, 140, 151). Moreover, it sufficiently abolished blister formation after passive transfer of PV-IgG (152, 171) or PF-IgG (174, 175) in mice.

There are strata of protein kinase cascades functionally subordinate to p38MAPK (176). Mitogen-activated protein kinase 2 (MK2) regulates several cellular activities such as actin remodeling (177), an event which can be correlated to PV pathogenesis. Phosphorylation of MK2 has been detected upon p38MAPK activation by PV-IgG (173), the inhibition of which was protective both *in vitro* and *in vivo*. Rho A is crucial to maintain a strong keratin association with desmosomes, enhance cortical actin filaments, stabilize cytoskeletal bound Dsg1 and Dsg3, and was found to be inactivated following PV-IgG and PF-IgG in p38MAPK-dependent manner (131). Besides, toxin-mediated inhibition of Rho GTPases recapitulated the PV-IgG-induced suprabasal blistering in human skin (132).

**Figure 3:**
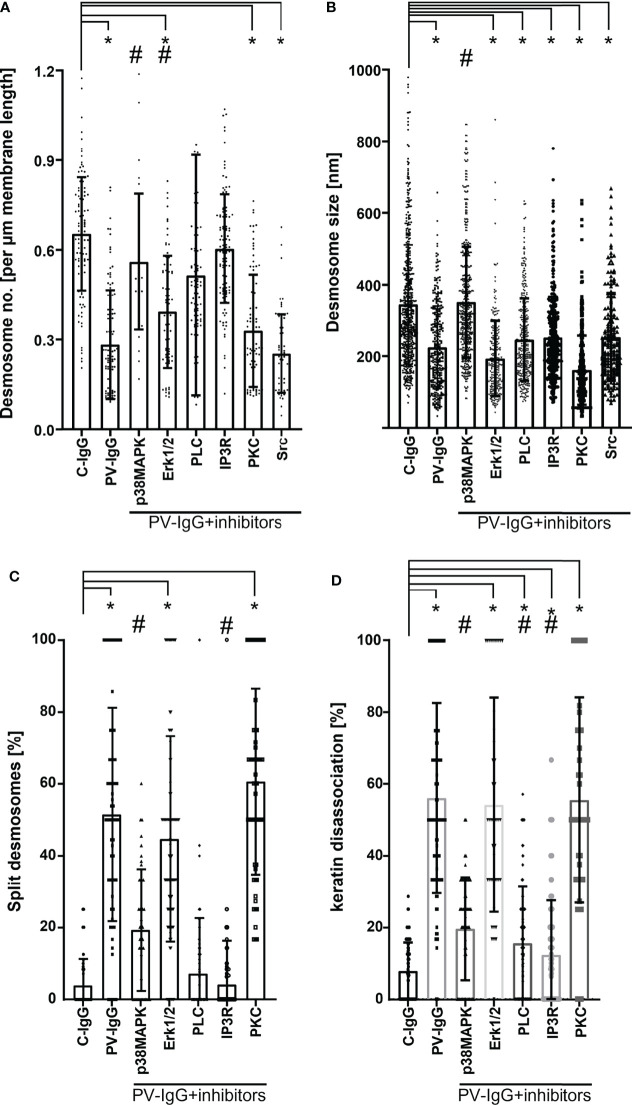
Ultrastructural quantification of desmosomes. **(A)** Desmosome density expressed in number of desmosomes per µm membrane length. Only desmosomes along clearly delineated cell borders of basal cells were considered. **(B)** Desmosome size measured along the linear length of the plaques and expressed in nm. **(C)** Percentage of split desmosomes both in acantholytic and non acantholytic areas. **(D)** Percentage of keratin dissociation from desmosomal plaques. Each data point represents individual desmosomes for **(B)**, and the average per electron micrograph for **(A, C** and **D)** (n= 3-5 for each pathway. *p < 0.05 vs. control, #p < 0.05 vs. PV-IgG). Inhibitors used: SB202190 – p38MAPK inhibitor, Pp2 – Src inhibitor, Bim-X – PKC inhibitor, UO126 MEK (upstream of Erk1/2) inhibitor, U-73122 – PLC inhibitor, Xest (xestospongin) – IP3R inhibitor.

We employed human organ culture models to assess the role of p38MAPK in mediating the ultrastructural changes of desmosomes in PV pathogenesis and found that inhibition of p38MAPK abolished blister formation in epidermis but not in mucosa, indicating that p38MAPK is crucial for the mechanisms causing epidermal blisters but not mucosal erosions (32, 135). Interestingly, loss of desmosomes as well as all ultrastructural alterations of desmosomes, including reduction in size, splitting, and keratin filament dissociation, were averted by inhibition of p38MAPK (**Figures 3A–D**).

These results suggest that modulation of this signaling pathway would be effective in treating pemphigus patients. However, a clinical trial using a p38MAPK inhibitor was terminated because of dose-limiting hepatotoxicity and did not show therapeutic benefits (178). This observation is important because it reveals that not all signaling pathways that are sufficient to stabilize desmosomal adhesion are druggable in patients.

### ERK But Not PKC or Src Partially Regulates Desmosome Ultrastructure

The MAP kinase ERK, a downstream signal effector of EGFR, was phosphorylated upon activation by PV-IgG and specific inhibition of which prevented acantholysis in cell cultures (48, 158, 161). Interestingly, ERK was activated in the presence of Dsg1 autoantibodies only (48, 162) and its specific inhibition was protective against PV-IgG or PF-IgG in cultured keratinocytes (48). Dsg1 is required to suppress ERK1/2 signaling to promote keratinocyte differentiation in the suprabasal epidermis where it is strongly expressed (159) by interacting with ErbB2 binding protein (Erbin2) (179). Mek1 inhibition, an upstream target of ERK, was sufficient to avert epidermal blistering in human skin *ex vivo* (21, 134) and reduce in number of desmosomes primarily along the basal-suprabasal interface but did not prevent desmosome splitting and keratin detachment from desmosomes (**Figures 3A–D**) (134).

On the other hand, for PKC and Src, the human *ex vivo* skin model yielded results different from what was found in cultured keratinocytes *in vitro* or for *in vivo* mouse models. PKC has been shown to be involved in signaling, Dsg3 depletion, loss of cell adhesion, and blister formation in cultured keratinocytes as well as in mice (48, 110, 147, 156, 180). Nevertheless, PKC inhibition did not ameliorate skin blistering in the human skin model and failed to modulate ultrastructural alterations (181) (**Figures 3A–D**). This discrepancy may be caused by the fact that several PKC isoforms exist which might be involved differently in both desmosome assembly and disassembly (22, 64). Src regulates desmosome assembly *via* interaction of Dsg3 with E-cadherin (169, 182). Reduced phosphorylation of Src was detected along with decreased Dsg3 expression in basal keratinocytes surrounding blister cavities, linking this pathway to PV pathogenesis (182). Inhibition of Src prevented cell dissociation (48, 59, 183, 184) and abrogated manifestation of skin lesions in mice but not in human skin culture where it was also not sufficient to modulate ultrastructural alterations of desmosomes (**Figures 3A–D**) (59). Since Src, similar to PKC, appears both to participate in desmosome assembly, a process which may require cortactin (59, 162), and to cause loss of desmosome adhesion in response to PV-IgG, this may explain the discrepancy between *in vivo* data in mice and *ex vivo* studies in human skin. Alternatively, the role of Src in PV pathogenesis may depend on the autoantibody profile of patients because the PV-IgG fraction used in human skin included higher levels of antibodies targeting Dsg1. It was shown that Src-mediated EGFR activation is associated with anti-Dsg3 autoantibody-mediated signaling rather than with signaling caused by autoantibodies against Dsg1 or Dsc3 (162, 185). Therefore, it is possible that Src inhibition may be effective in treating some PV patients but not others.

However, for all studies using electron microscopy evaluation of human epidermis, the limitation is that they are feasible by using a very limited number of patients’ IgG fractions only. Thus, no preliminary dose-response characterization is possible, and it cannot be ruled out that, with autoantibody samples from other patients or using higher concentrations of pharmacological inhibitors, a protective effect may be found for other signaling molecules as well. Therefore, negative data must be taken with extreme care. On the other hand, if a protective effect under all conditions and in all models, including human skin, is observed for modulation of a specific signaling pathway, this is a strong indication for a potentially interesting treatment paradigm.

### PLC Mediates Desmosomal Adhesion by Maintaining Keratin Anchorage to Desmosomes

PV-IgG augments intracellular Ca^2+^ and inositol 1,4,5-trisphosphate (IP_3_) levels (186) *via* activation of Phosphoinositid-Phospholipase C (PLC) (147) leading to cell dissociation. IP3, *via* activation of IP3 receptor (IP3R), causes Ca^2+^ release, which in turn activates PKC (187). Inhibition of PLC was protective against the dyscohesive effect of PV-IgG in cell culture (147), *in vivo* (157), and in human skin explant (133, 136). Interestingly, specific inhibition of PLC and Ca^2+^ signaling significantly ameliorated keratin dissociation and desmosome splitting (**Figures 3A–D**) (133).

### Signaling Pathways Regulate Desmosome Turnover

It is well established that pemphigus is a desmosome turn-over disease (188) because the signaling pathways involved interfere with different steps of desmosome assembly and maturation on one hand, and desmosome internalization and disassembly on the other. The interesting question is whether the ultrastructural analyses as outlined above allow to allocate signaling pathways to specific steps of desmosome turnover. Loss of desmosomes, which was caused by mucocutaneous PV-IgG but not by mucosal dominant PV-IgG and AK23, is the ultimate consequence of a dysbalance between assembly and disassembly. Thus, depletion of extradesmosomal Dsg molecules, which serve as a pool for incorporation into existing desmosomes, may account for this phenomenon (109, 189). Similarly, it was shown that following depletion of Triton-soluble extradesmosomal Dsg3, Triton insoluble Dsg3 was reduced as well (109). This brings up the important question of whether the latter Dsg3 molecules were derived from desmosomes, which would be characterized best as desmosome disassembly, or whether this is just the consequence of impaired assembly. Experiments showing that Dsg3 internalization following autoantibody binding is a coordinated process involving endocytosis are compatible with both interpretations (111, 170, 171, 190–193).

Recently, it was reported in Madin–Darby canine kidney (MDCK) cells that hepatocyte growth factor (HGF) induces internalization of intact desmosomes with no alteration in desmosome size and composition, which was interpreted as desmosome internalization (194). As outlined above, the situation in pemphigus is different and more complex. PV-IgG cause significant shrinkage of remaining desmosomes (**Figure 3B**). In addition, transmission electron microscopy and structured illumination microscopy investigations have revealed that desmosomes in neonatal mice were split prior to internalization (124) and were present at cell surfaces surrounding blister cavities in patients’ lesions (129, 138, 195–198) and human *ex vivo* skin (**Figure 3C**) (181). These data show that desmosome splitting occurs when desmosomes are weakened due to an imbalance of desmosome assembly and disassembly which is facilitated by mechanical stress (198). In parallel to desmosomes with reduced size, double-membrane structures together with desmosomes which were uncoupled from keratin filaments were also observed (135), indicating that desmosome internalization in skin occurs similar to MDCK cells, which represent a single-layer epithelium. However, internalized desmosomes were rare and thus could not be evaluated in quantitative terms.

These results show that desmosome disassembly and internalization may be dependent on both cell type and stimulus. For both events, uncoupling of Dsg molecules from the cytoskeleton was shown (**Figure 3D**) (32, 133, 181, 194). It remains unclear to which extent mechanical destabilization of desmosomes resulting in desmosome splitting under force results from desmosome shrinkage or keratin filament dissociation. The data from ultrastructural evaluation of desmosomes indicate that inhibition of p38MAPK was the only pharmacologic intervention to rescue desmosome size (**Figure 3B**). On the other hand, both p38MAPK and PLC/Ca^2+^ were effective to modulate keratin dissociation and splitting of desmosomes, which can be interpreted that cytoskeletal anchorage is sufficient to prevent desmosome splitting. The data are in line with experiments showing that phosphorylation of Dp by PKC, which is activated downstream of PLC/Ca^2+^, in PKP1-dependent manner regulates intermediate filament anchorage, and thereby causes desmosome hyperadhesion by trapping of desmosome components (199–201). In response to PV-IgG, PKC-mediated Dp phosphorylation reverts the hyperadhesive state and causes loss of desmosome adhesion (180, 202). Another conclusion is that steric hindrance alone cannot explain these ultrastructural alterations observed in patient lesions and *ex vivo* skin model because this mechanism would cause split desmosomes with intact size and keratin filament anchorage.

Taken together, p38MAPK and PLC/Ca^2+^/PKC were shown to be involved in depletion of Dsg1 and Dsg3 (110, 112, 136), as well as in keratin filament dissociation (133, 135), indicating that these signaling pathways are important for disturbed desmosome assembly as well as for desmosome disassembly and internalization in pemphigus pathogenesis. For Src and ERK, it remains less clear which mechanisms are involved in loss of keratinocyte adhesion. It is likely that they contribute to the same mechanisms impairing desmosome turnover like p38MAPK and PLC-mediated signaling but may be less central for these events.

### Concluding Remarks

The ultrastructural analysis in human epidermis revealed that for all signaling pathways where pharmacologic modulation was protective, an ultrastructural correlate in desmosomes was found (**Table 1**). Based on this, we conclude that investigations on desmosome composition are helpful to advance our understanding of the regulation of desmosome turnover and to ultimately decipher a signaling pathway which may be druggable in patients as an additional line of therapy until depletion of pathogenic autoantibodies is effective and to reduce potential side effects.

**Table 1:** Protective effects of signalling pathway modulation.

Protective Effects of Signaling Pathway Modulation							
	*In vitro* (dissociation assay)	*In vivo* (passive transfer)	*Ex vivo* (human epidermis)	*Ex vivo*ELMI:	*Ex vivo* ELMI:	*Ex vivo*ELMI:	*Ex vivo*ELMI:
PV-IgG +inhibitor	Loss of adhesion	Blisters	Blisters	Desmosome Loss	Desmosome shrinkage	Split desmosomes	keratin retraction
P38MAPK	+	+ (Berkowitz, 2006)	+ (Egu 2017)	+ (Egu 2017)	+ (Egu 2017)	N.D.	+ (Egu 2017)
ERK	+	N.D.	+ (Egu 2019) (Burmester 2020)	+(Egu 2019)	+ (Egu 2019)	- (Egu 2019)	- (Egu 2019)
PLC	+	+ (Sanchez- Carpintero 2004)	+ (Schmitt 2021)	- (Egu 2022)	- (Egu 2022)	- (Egu 2022)	+ (Egu 2022)
PKC	+	+ (Sanchez- Carpintero 2004) (Spindler 2011)	- (Egu 2019)	- (Egu 2019)	- (Egu 2019)	- (Egu 2019)	- (Egu 2019)
Src	+/-	+ (Pretel 2009) (Kugelmann 2019)	- (Kugelmann 2019)	- (Kugelmann 2019)	- (Kugelmann 2019)	- (Kugelmann 2019)	N.D.

Signalling pathways were selected when compelling evidence was obtained from in vitro studies by numerous groups as outlined in the text and ultrastructural ex vivo data were available. It can be concluded that for all signalling pathways critical in pemphigus pathogenesis, an ultrastructural correlate in desmosomes was found in human epidermis. ND, Not Done.

Electron microscopy is a gold standard method to define ultrastructural alterations of desmosomes in pemphigus patient lesions (138) and to investigate the underlying mechanisms of a given signaling molecule in human skin, the role of which has been suggested by studies in cultured keratinocytes or mouse models. By this approach, mechanisms occurring in cell culture and mouse skin, but not in human epidermis, such as apoptotic cell death, also can be evaluated (138, 203). In the long run, super-resolution microscopy will be applied to study the pathogenesis of pemphigus in more detail as has been started already (170).

## Author Contributions

DE: concept, original draft, and revision. JW: concept and revision. TS: figure design, schematic drawing. DE and JW equally contributed to the article. All authors read and approved the final manuscript.

## Funding

This work was supported by the DFG FOR2497 (PEGASUS) grant to JW.

## Conflict of Interest

The authors declare that the research was conducted in the absence of any commercial or financial relationships that could be construed as a potential conflict of interest.

## Publisher’s Note

All claims expressed in this article are solely those of the authors and do not necessarily represent those of their affiliated organizations, or those of the publisher, the editors and the reviewers. Any product that may be evaluated in this article, or claim that may be made by its manufacturer, is not guaranteed or endorsed by the publisher.
